# Construction Site Noise Exposure Assessment Using Binaural Measurements and Analysis

**DOI:** 10.3390/safety10040092

**Published:** 2024-10-30

**Authors:** Nikolina Samardzic, Virginia Best, Christian Hammond, Aslihan Karatas, Behzad Esmaeili, Haitham Sarsam

**Affiliations:** 1 Department of Engineering Technology, Lawrence Technological University, Southfield, MI 48075, USA; 2 Department of Speech, Language & Hearing Sciences, Boston University, Boston, MA 02215, USA; 3 Department of Civil, Materials, and Environmental Engineering, University of Illinois at Chicago, Chicago, IL 60607, USA; 4 School of Industrial Engineering, Purdue University, West Lafayette, IN 47907, USA; 5 O’Brien Construction, Bloomfield, MI 48302, USA

**Keywords:** noise exposure assessment, binaural measurement and analysis, sound impulsiveness, kurtosis

## Abstract

Acoustic measures of construction site noise are important for hearing loss prevention and safety. This work examines noise exposure assessment using binaural measurements to pave the way for more accurate noise characterization and hearing loss prevention in loud workplaces. Recordings were made over three days on a construction site, and noise exposure was estimated using state-of-the-art methods (a shoulder-worn dosimeter) and binaural measurements (microphones at each ear). For the binaural assessment, noise exposure was quantified at each ear to identify the higher- and lower-exposure ears, and the assessment incorporated kurtosis, a statistical measure that quantifies impulsiveness of the noise. The impulsiveness of the noise of four construction tools was also assessed. For this set of measurements, traditional hearing loss risk assessment consistently underestimated the noise exposure relative to binaural assessment. Moreover, the binaural measurement method exposed multiple cases of asymmetric noise exposure that are not detectable using the traditional method. Overall, there are clear benefits to assessing risk using binaural measurements and more detailed analysis methods.

## 1. Introduction

Approximately 51% of construction workers are exposed to hazardous noise [1], and 25% of noise-exposed construction workers develop hearing loss [2]. Hearing loss is the most common occupational disease in the US [3]. Construction workers are 40% more likely to have a hearing impairment and 27% more likely to suffer from tinnitus than workers in occupations with low noise exposure levels [4,5]. Hearing loss correlates with a higher risk of depression, social isolation, and cognitive decline [6].

Accurate assessment of noise exposure on construction sites is crucial for reducing instances of hearing loss. The most common occupational noise exposure measurement devices are single-channel dosimeters, worn on the shoulder, and sound level meters (SLMs) typically placed at convenient locations close to workers. Previous studies analyzed construction workers’ noise exposure using these devices to quantify the sound pressure and calculate the daily noise dose as recommended by OSHA [7] or NIOSH [8] and have contributed to controlling and mitigating occupational noise risks. However, single-channel dosimeters and SLMs do not capture complex sound characteristics that can affect how hazardous an exposure is. For example, impulsive noise exposure may result in the same calculated noise dose as another less dangerous steady-state noise exposure. This lack of detail in current noise measurements may increase the risk of excessive noise exposure at construction sites. Additionally, current sound measurement devices cannot be used to assess asymmetric sound exposure. Asymmetric hearing loss is a common benefit claim for noise-induced hearing loss cases and is often attributed to occupational noise exposure [9]. A relatively simple way to address both of these issues and improve the accuracy of noise exposure assessment is to use binaural measurements.

Previous studies have demonstrated that the effects of dosimeter microphone position can be large [10]. Although the top of the shoulder is historically considered an optimal position for a dosimetry microphone, the differences in sound pressure level among on-body locations can vary up to 15 dB [11]. This problem is even more relevant for impulse noise, where head shadowing and pinna resonances can strongly affect the high-frequency content of the energy delivered to the eardrum. The ideal microphone placement for personal dosimetry is in the ear since it requires very few assumptions about the activities of the individual. Recent in-ear noise dosimetry studies, involving miniature microphones placed inside the ear canals, have demonstrated the ability to monitor daily noise levels with such a system [12–14]. However, these studies did not consider how measurements from the two ears could be combined and related in a meaningful way to hearing loss risk. In addition, in-ear dosimetry may interfere with standard hearing protection, situational awareness, and comfort, making it impractical for long-term use. Near-ear dosimetry can provide a good compromise of fidelity and practical use.

Sound pressure level (SPL), expressed in decibels (dB), is the standard acoustic evaluation metric using noise dosimeters and SLMs. However, Kardous [15] found that SPL-based noise dose measurements are not highly correlated with the hearing damage observed in practice for participants exposed to high-level impulsive sounds. Further, they found that construction workers may not regard hammer noise as dangerous because of the misconception that only extreme loudness leads to damage. These findings raise an important issue regarding noise dose calculation. Specifically, a more dangerous impulsive noise exposure may result in the same calculated dose as another less dangerous steady-state noise exposure using traditional SPL-based metrics. Commercially available noise dosimeters do not perform properly in impulsive noise environments because they suffer from instrumentation limitations and lack metrics that characterize impulsive noise [12,15]. As a result, while these systems provide a time-weighted average SPL that has received wide acceptance as a damage risk metric for continuous-noise environments, they may not be adequate for predicting hearing damage from complex or impulsive noise [11,16,17].

Yang [18] obtained binaural measurements of typical construction noises, relating the physical and psychoacoustic properties of the noises (e.g., loudness fluctuation strength, roughness, and sharpness) to workers’ perceptions of the noises and their hearing and ability to communicate. Their analysis methods used the arithmetic average of the data from the two ears, or chose one ear arbitrarily, and thus did not consider asymmetries. In another recent work, Zhang [19,20] used kurtosis to improve estimates of noise exposure in relation to hearing loss from industrial noises in manufacturing. Kurtosis is a statistical measure that quantifies the impulsiveness of a noise. The A-weighted 8 h noise exposure level was adjusted according to the kurtosis, with the adjustment equivalent to adding a penalty. Kurtosis has not yet been used in the assessment of noise on construction sites.

The current study, based on a preliminary study [21], aimed to demonstrate the potential advantages of a binaural assessment method that quantifies noise exposure at each ear to identify the higher- and lower-exposure ears and incorporates kurtosis. The study had three specific objectives:

Objective 1: Test the hypothesis that binaural measurements and analysis can reveal (a) clinically significant differences (>1 dB) relative to traditional SPL-based single-channel dosimeter noise exposure assessment; and (b) asymmetrical noise exposure (≥1 dB between higher- and lower-exposure ears).

Objective 2: Calculate the correlation between single-channel dosimeter SPL measurements and two-channel binaural SPL measurements with and without kurtosis adjustment.

Objective 3: Compare the noise impulsiveness and hazardousness of common construction tools using the kurtosis calculations.

## 2. Research Methodology

### 2.1. Measurement Equipment

This study’s measurement equipment included the BK Type 4448 dosimeter (a monaural system) and the HEAD Acoustics SQobold binaural data acquisition (DAQ) system. The dosimeter sampling was at 32 kHz, and the SPL was stored after every minute and, as such, available for analysis. The raw sampled time history was unavailable for analysis as it could not be stored by the device. The dosimeter SPL data were exported and viewed offline using the Work Noise Partner software, version 1.6.3. The SQobold is a mobile four-channel data acquisition system that allows for the recording, analyzing, and playback of recorded signals. Alongside the headset connector, it has two BNC inputs, a pulse input, GPS, and allows for the connecting of a USB camera for additional data documentation. It weighs about 1 lb and fits comfortably into a coat pocket. The 64 GB onboard memory, as well as the SQobold sampling at 48 kHz for two channels (left and right ear), allows for at least 10 h of recording at a time. This measurement duration and high sampling frequency results in a large amount of data in the time domain that can be partitioned and analyzed offline. The offline analysis of binaural measurements included SPL and kurtosis calculations for each ear in the ArtemiS SUITE software, version 15.5.

**Figure 1.**
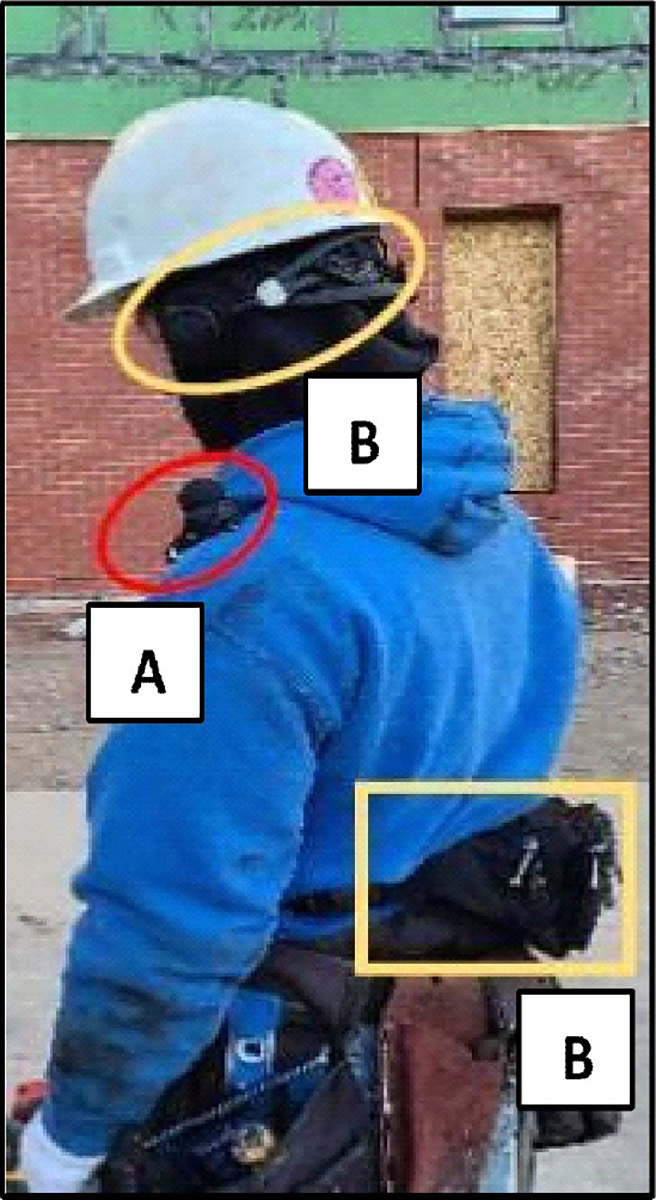
Noise exposure measurement setup with SQobold binaural headset and DAQ system (yellow, B) and traditional dosimeter method (red, A).

Figure 1 illustrates an example location of the dosimeter and the binaural devices. Depending on the handedness of the worker, the dosimeter was placed on the shoulder of the side of the working hand, presumably closer to the noise sources throughout the day. However, if the work analysis conducted before the measurements revealed that the work would involve using a particularly preferred shoulder, for example, for carrying materials throughout the day, the dosimeter was placed on the opposite shoulder.

The binaural measurement configuration consisted of an adjustable headband on the BHS II headphone/microphone unit. For this study, the headband was placed above the adjusting knob of a hard hat for stable positioning and a comfortable fit (Figure 1). Earplugs or other in-ear hearing protective devices would fit unobtrusively under the headphones. The low-weight adjustable earpieces, equipped with two ICP^®^ microphones with removable windscreen, were specifically designed for environmental/exterior noise measurements. They were calibrated with a microphone calibrator via a ¼-inch adaptor prior to all the measurements. The SQobold DAQ system was carried around the waist in a pouch (a “fanny pack”). This provided protection from exposure to the dusty and dirty environment on the construction site. The most convenient location for the pouch was determined by the worker/volunteer. The wire connecting the headset to the DAQ system was covered by the workers’ clothes and not exposed to the work environment.

### 2.2. Study Participants and Measurement Methods

The construction noise measurement site was a multi-family unit project in Michigan. The floor plan and the work area of the construction workers, highlighted in red, is shown in Supplementary Materials. Three construction workers participated in monaural and binaural sound measurements on each of the three work days. On the first two measurement days, the workers consisted of two carpenters and a plumber. On the third day, the workers consisted of three carpenters. One carpenter participated on all three days. Prior to the measurements, work analysis with job and task information planned throughout the day was obtained from the workers. The worker activities were also observed and recorded along with comments from the workers obtained at the end of each day regarding their perception of the levels and loudness of the noise experienced throughout the day. The work consisted of wall framing for the carpenters and plumbing installation for the plumber. The worker locations varied in a range between 1 and 10 feet from the exterior walls. The tools used by the workers were a Paslode F350-S nail gun, a DeWalt DWHT51001 hammer, a DeWalt DCD791 drill, and a DeWalt DCS382B saw. The sound power levels (Lw) for the nail gun, drill, and saw were specified at 120 dBA, 86 dBA, and 94.5 dBA, respectively.

Acoustic measurements were obtained for the duration of an 8 h shift on each day. This measurement strategy was selected because it provided the complete raw data measurements needed for a comprehensive acoustic assessment. The measurements made use of three sets of dosimeters and SQobold binaural DAQ systems. Approximately 960 gigabytes of data in total were recorded over the three days. The data were then organized with a detailed file-naming convention and prepared for post-processing.

**Figure 2.**
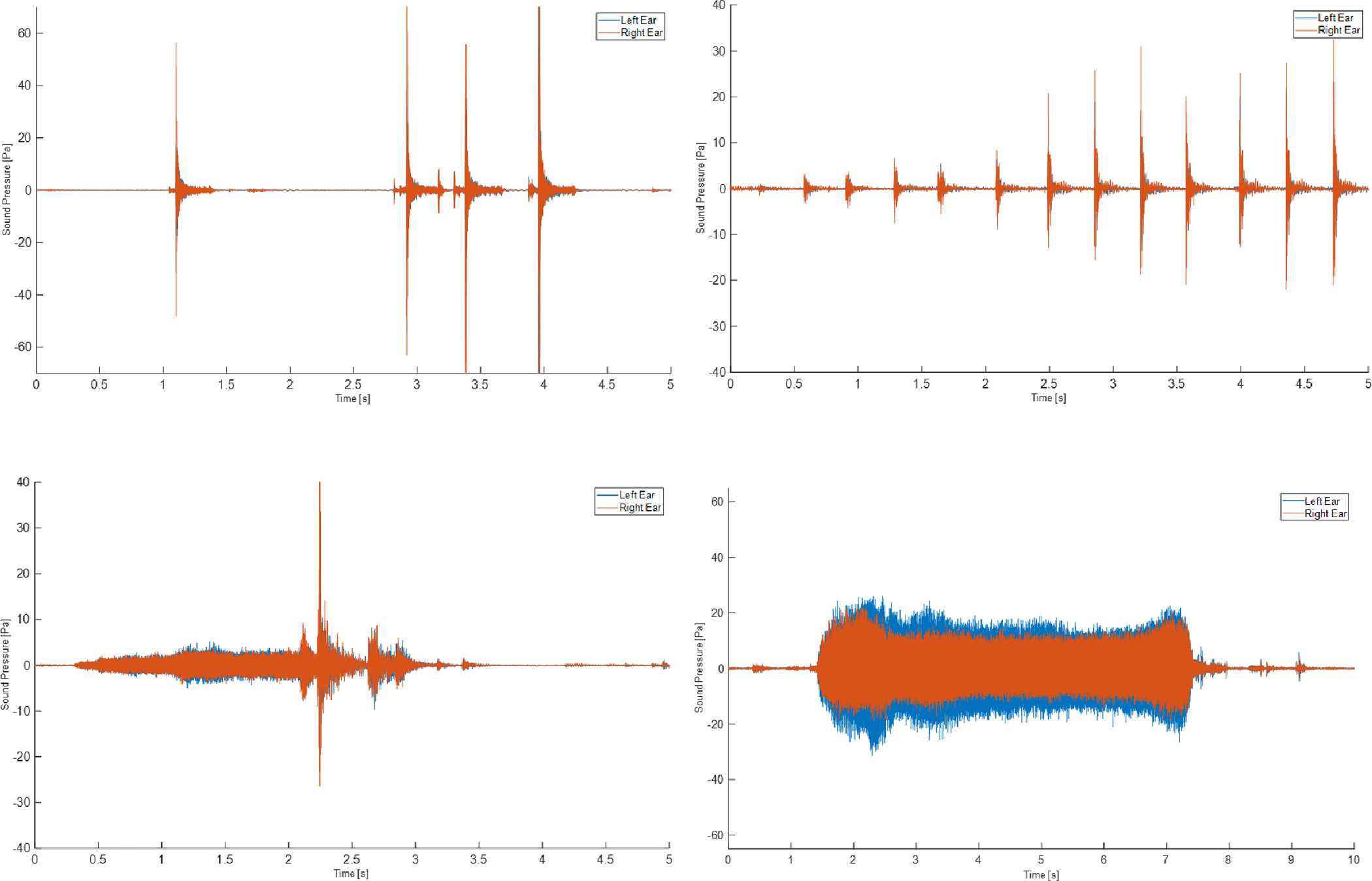
Typical time histories of measured sound pressure signals from various events: nail gun (**top left**), hammer (**top right**), drill (**bottom left**), and saw (**bottom right**).

First, for binaural measurements, piecewise analysis of the recorded signals was performed, where the analyzed “pieces” or selected time segments of the recorded signals were associated with particular tasks or jobs. Specifically, the segments were associated with impulsive events from activities from impulsive events, including activities from the four construction tools (nail gun, hammer, drill, saw) used throughout the work days. In total, there were 166 segments available for analysis (57 nail gun events, 47 hammer events, 30 drill events, 17 saw events, and 15 impulsive events not associated with any tool or task). For the selected segments, the background noise SPLs on the construction site were significantly lower (<10 dB) compared to the SPLs of the impulsive events under consideration. The average duration of the segments containing impulsive events was 15 s (standard deviation 21 s). The extraction and labeling of segments from the recordings were performed manually by the student assistants. As a quality-checking mechanism, multiple student assistants went through the recordings to confirm that the extracted segments and tool labeling agreed. Figure 2 shows typical time histories of measured sound pressure signals from various events.

For dosimeter measurements, the segmenting process could not be conducted in the same way because dosimeter SPLs were available only after every one-minute segment. Instead, 166 dosimeter readings were identified, corresponding to one-minute segments that contained each of the impulsive events previously identified from the binaural measurements. This allowed for a comparison of the dosimeter (monaural) and left and right ear (binaural) SPLs for overlapping time periods. The time stamps of the dosimeter segments and their SPLs were identified offline using Work Noise Partner software and matched with the time stamps of the binaural recordings identified using ArtemiS SUITE software, version 15.5.

## 3. Analysis Methods

### 3.1. SPL Calculation

The traditional noise exposure assessment is based on the SPL calculation. The SPL calculation utilizes the root mean square value of the sound pressure measurements (*p*) over the selected time frame, associated with a job, task, shift, or specific time duration of a noise event of interest. The reference pressure (*p*_*ref*_) is 0.00002 Pa. The exposure level calculations based on SPL assessment utilized the methods described in ISO 9612 [22]. The calculation was performed using ArtemiS SUITE software, version 15.5.

### 3.2. Kurtosis Calculation

Kurtosis is a statistical measure that quantifies the impulsiveness of the noise; the higher the kurtosis value, the stronger the noise impulsivity. The kurtosis (β) calculation, performed using ArtemiS SUITE software, version 15.5, involves the ratio of the fourth moment of a signal to the squared second moment of the same signal, whereby N specifies the number of samples in the integration interval:

$$\beta =\frac{\frac{1}{N}\sum_{n=0}^{N-1}\;s(n{)}^{4}}{{\left[\sum_{n=0}^{N-1}\;s(n{)}^{2}\right]}^{2}}.$$

In our analysis, kurtosis was calculated using 50 ms integration windows with a 50% overlap, and a maximum kurtosis value was extracted for each recording segment. In this study, the kurtosis adjustment method suggested by Zhang [19] was used, i.e., adding a correction value (Δ, in dB) to the measured SPL, using the following formula:

$$\Delta =6.5\left(\frac{\beta }{3}\right).$$

**Figure 3.**
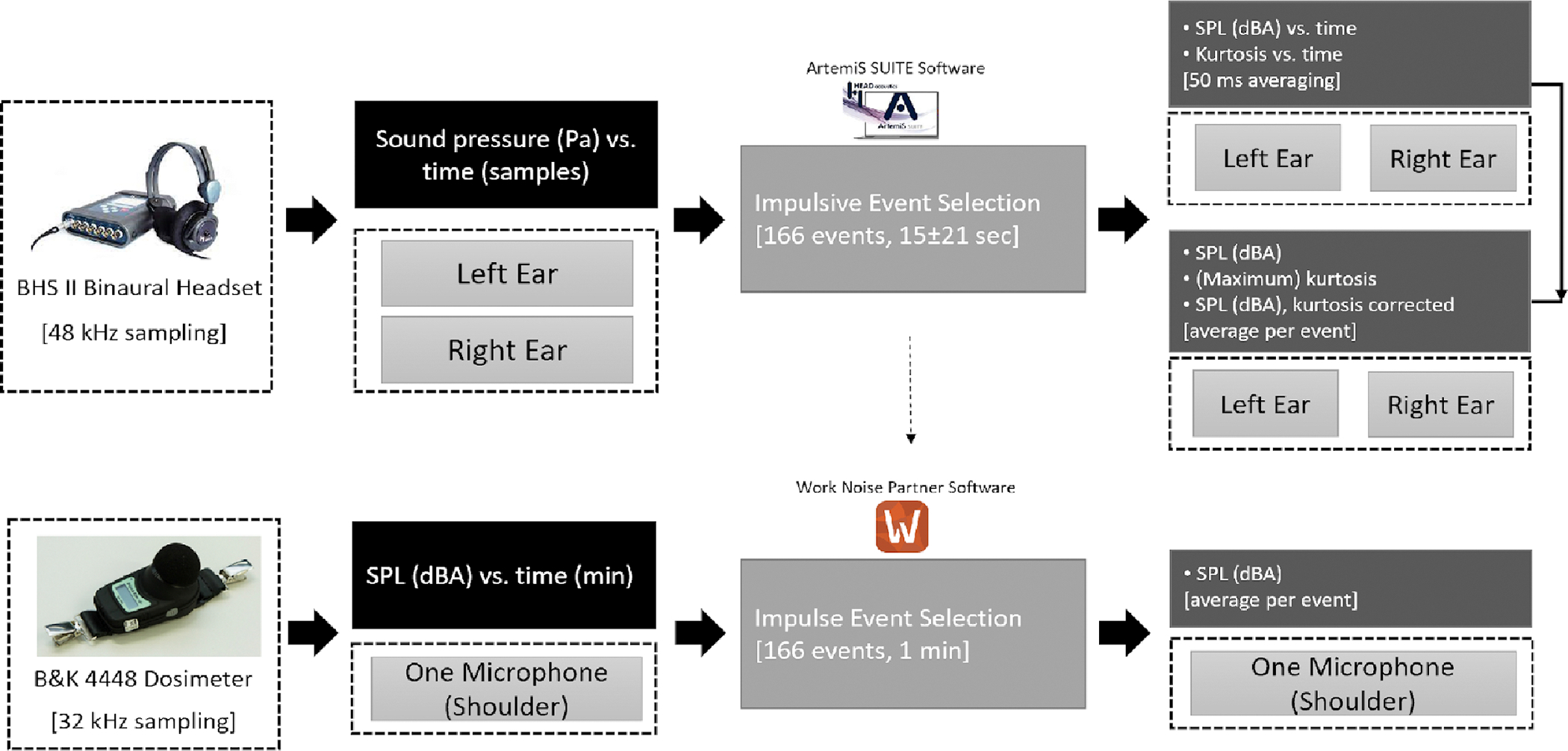
Measurements and SPL and kurtosis analysis overview.

An overview of the measurements and SPL and kurtosis analysis is shown in Figure 3.

## 4. Results

The descriptive statistics for the 166 samples considered in this study are summarized in Table 1. All quantities in Table 1 are normally distributed based on the one-sample Kolmogorov–Smirnov test (*p* < 0.05).

**Figure 4.**
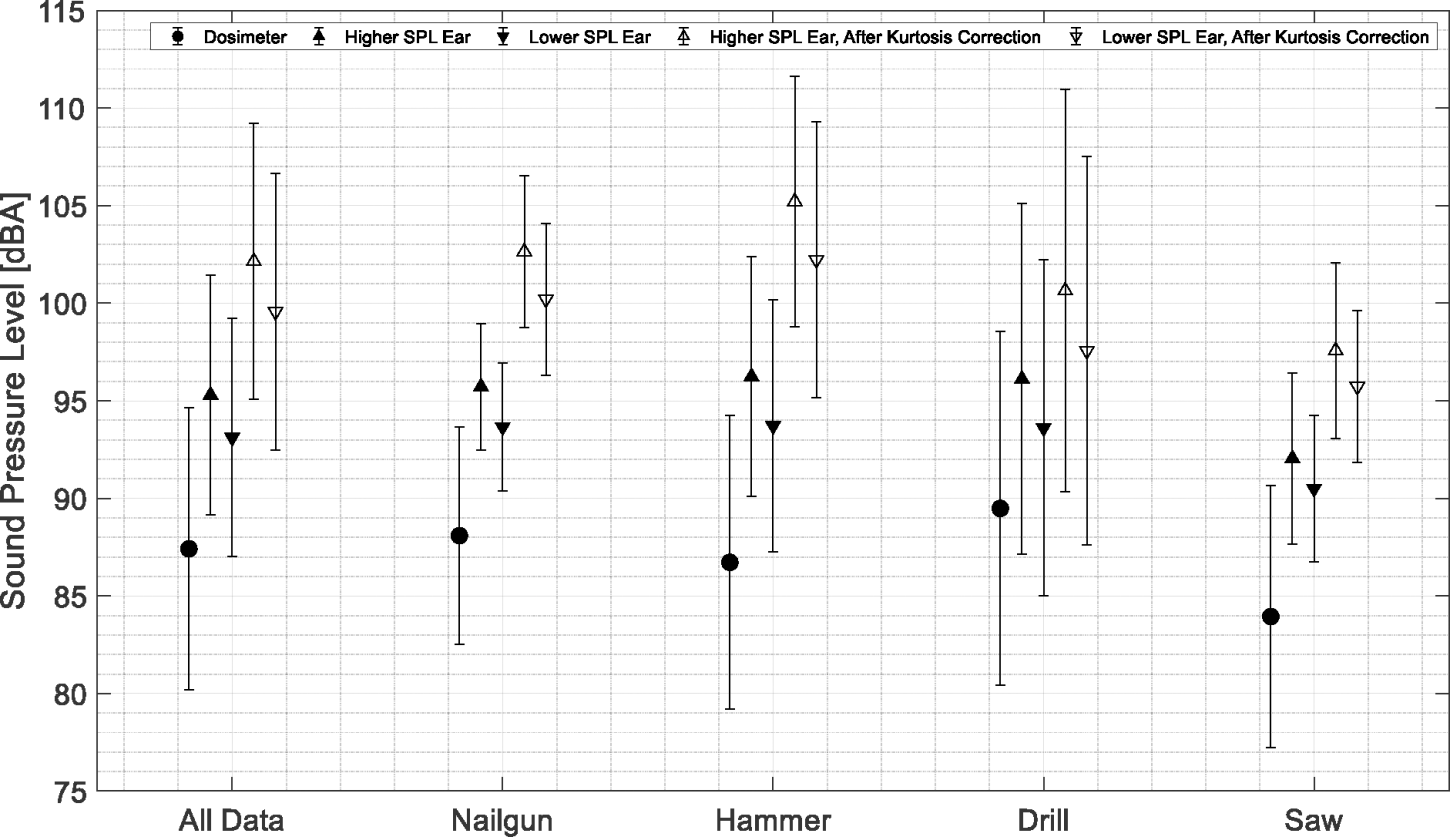
SPL (dBA) measurements using a dosimeter and binaural headset and kurtosis-corrected SPL (dBA) for each ear for all data (left) and with the data categorized according to operating tool.

For Objective 1, we compared the SPLs obtained from the single-channel dosimeter and from the two-channel binaural measurements. A *t*-test was used to compare the mean values of the datasets from the dosimeter and binaural measurements. The null hypothesis was that the mean difference between the datasets was 1 dB. The statistical analysis yielded the results in Table 2. The average difference between the higher-SPL ear and the dosimeter measurements was 7.9 dB, with a standard deviation of 6.0 dB, and the null hypothesis was rejected, suggesting that the difference is clinically significant. A *t*-test was used to compare the average SPL from the left ear and the right ear and the higher-SPL ear and the lower-SPL ear to quantify asymmetrical noise exposure. The average SPL difference between the left ear and the right ear was 0.92 dB, with a standard deviation of 2.5 dB, which is not a clinically significant effect. The average SPL difference between the higher-SPL ear and the lower-SPL ear was 2.2 dB, with a standard deviation of 1.5 dB, which is a clinically significant difference, and we would argue that it is a more accurate measure of asymmetry than the left–right difference, which does not account for the fact that the higher-SPL ear is not necessarily the same ear for all 166 samples. These key points are illustrated in Figure 4 (leftmost cluster of points).

For Objective 2, the analysis was based on evaluating if single-channel dosimeter SPL measurements are correlated with two-channel binaural SPL measurements with and without kurtosis adjustment. To achieve this, a correlation analysis was performed. Dosimeter measurements were significantly (*p* < 0.001) correlated with both higher and lower ear SPL and with kurtosis-adjusted higher and lower ear SPL. However, the relationships were rather weak, with linear correlation coefficients (*r*) of 0.60, 0.62, 0.54, and 0.56, respectively. Given that these measurements were taken at similar moments under identical conditions, these weak correlations reinforce the point that the measurement method has a strong influence on estimates of noise exposure.

To address Objective 3, Figure 4 shows the data categorized according to the specific operating tool being used. Although the overall values differ across the categories, the trends within each category are similar to those for the combined data (leftmost cluster of points) in terms of the difference between dosimeter and binaural measurements and the SPL differences across ears. As expected, the kurtosis adjustment varies depending on the sound impulsiveness associated with a particular tool. Previously, kurtosis was used to assess the hazardousness of sound [19,20]. In our study, the hammer sound resulted in the greatest kurtosis adjustment and the highest adjusted SPL overall, suggesting it is more hazardous than the other three types of sound.

Kurtosis analysis highlighted the fact that noise hazardousness is not always related to higher SPL. For example, for 16/166 (9.6%) of the events/measurements, the lower-SPL ear became the higher-SPL ear after kurtosis correction (and vice versa). For these 16 events, the average absolute SPL difference between the higher-SPL ear (as measured) and the higher-SPL ear after kurtosis correction was 1.17 dB, with a standard deviation of 1.4 dB.

## 5. Discussion

The SPL results comparisons in this study resulting in differences of at least 1 dB were considered to be significant. The justification for this criterion is the fact that a 1 dB underestimation in SPL results in underestimation of the noise dose, ranging from 15% using the OSHA noise dose calculation method to 26% using the NIOSH noise dose calculation method, as presented in Table 3. The daily, permissible noise dose calculation is based on the noise exposure level and the duration of the exposure. Each noise level increase of 3 dB (NIOSH [8]) or 5 dB (OSHA [7]) causes the noise dose to double. Any difference of at least 1 dB will be clinically significant. A 1 dB measurement error would mean that a worker may be overexposed to noise between 1 h (OSHA) and 1 h and 20 min (NIOSH). For steady-state noise levels, there is no difference between the doses calculated using the NIOSH and OSHA calculation methods, but in a highly fluctuating noise environment, such as a construction site, the difference is pronounced; the use of the OSHA calculation method puts construction workers at higher risk than workers exposed to similar average steady-state noise [23].

This study evaluated a binaural measurement system and analysis method designed to enable more-complete noise exposure assessments on construction sites. This method produced consistently higher noise estimates than the current single-channel dosimeter approach. The SPL obtained from the dosimeter measurements, averaged over one-minute timeframes, was compared to the SPL obtained from the two-channel binaural measurements, averaged over the duration of the impulsive events samples. A contributing factor to the lower dosimeter SPL was longer averaging in one-minute timeframes containing not only the shorter impulsive events but also capturing other background/steady/non-impulsive noise with lower SPL. An improvement to the current dosimeter technology would be a higher data storage capacity device capable of recording and continuously analyzing sampled data at shorter (milliseconds as opposed to minutes) intervals. This would allow for the accurate assessment of hearing risk from impulsive events through calculations of SPL, kurtosis, and any other relevant psychoacoustic or hearing risk metrics. Future similar dosimeter/single-channel and binaural/two-channel measurements could then be compared using measurement timeframes obtained at identical moments and under identical conditions.

Due to the fact that the higher-SPL ear was not the same ear (left or right) for all impulsive event exposures throughout the day, asymmetry was measured in the current study as the difference between the higher-SPL ear and the lower-SPL ear (as opposed to a difference between the averaged SPLs and the left and the right ears). This measurement is an improvement on the current noise exposure assessment, which is a calculation of noise dose based on average SPL sampled and recorded continuously throughout the day and obtained from a single dosimeter location or the left and/or right ear location. In an ideal system, the left and right ear measurements would be continuously compared, and the higher-SPL ear would be selected for the measured noise throughout the day and used in the noise dose calculation to capture the worst-case scenario for noise dose calculations.

The weak correlations between dosimeter/single-channel SPL and binaural/two-channel SPL (with and without kurtosis correction) indicate that the chosen measurement and noise assessment method has a strong influence on estimates of noise exposure. The binaural measurement system and analysis method was also able to reveal differences across sound types, as seen in significant (>1 dB) differences in kurtosis corrections between sounds emitted by different tools. This suggests that the binaural measurement system is capable of providing both accurate measurements of SPL and estimates of hazardousness to support more complete noise exposure assessments.

## 6. Limitations and Future Work

The findings in this study are based on a novel binaural data measurement and analysis method not previously presented in the literature. A potential limitation of the proposed binaural approach is that it requires significant acoustical expertise for analyzing the data compared to the traditional dosimeter approach, which presents a few standardized SPL-based metrics as readouts on the dosimeter. Another limitation of binaural measurements is the impact of environmental conditions, such as rain and wind, that may introduce unwanted sounds in the exterior noise measurements at one or both ear locations. A complete assessment of noise exposure on construction sites, where acoustic conditions can vary significantly depending on the day, the equipment used, and the stage of construction, would require a longer study period compared to the three days of measurements in the present study. Furthermore, future work should include the application of the proposed binaural assessment to other industries where noise exposure is significant, such as manufacturing and mining, in order to generalize the results of the current study.

The current study highlights the importance of accurately assessing noise exposure on construction sites, which is critical for reducing noise-induced hearing loss. However, this work is limited to occupational noise exposure assessment, and it does not consider situational awareness assessment such as auditory warning signal perception, which is also a critical safety consideration on a construction site. During construction work, alarm signals may be inaudible due to the noisy environment, increasing the risk of accidents such as being struck by objects, which is common on construction sites where trucks may back up or machines may rotate their arms. Additionally, in such noisy environments, emergency alarms may go unnoticed, jeopardizing workers’ ability to respond to evacuation warnings. Future work could utilize binaural recording methods to investigate warning signal detection and localization. Binaural recordings from construction sites offer a unique opportunity for research on this important topic. Specifically, binaural recordings maintain all spatial information of the sound field and allow for binaural replay of the recorded signals over headphones to recreate the perception of being present in the original sound field; this cannot be accomplished with recordings based on a monaural microphone (ISO TS12913–2 [24]). Leveraging this capability, future work could utilize binaural recording methods to investigate warning signal audibility and localization and to evaluate or model the performance of hearing protection devices.

Future work may also involve exploring psychoacoustic metrics, such as loudness, to quantify hearing risk on construction sites. Such metrics were previously used for characterizing construction noise; however, their relation to hearing risk is unknown. For example, Lee and Kim [25] conducted noise measurements with a microphone located 10 m from the construction machine and reported a loudness range of construction noises from fifteen machines between 21.8 and 98.7 sones. Lee et al. [26] found that the annoyance rating scores of individual and combined construction noises increased with increasing loudness; the effect of loudness on the perception of annoyance was much greater than the effects of the other factors of sharpness, roughness, and fluctuation strength. Further research is required to assess occupational noise from a wider variety of tools and different operating conditions and to determine whether psychoacoustic metrics such as loudness can provide further insight into the potentially damaging effects of noise compared to physical measures such as SPL, as well the difference in the metric values (i.e., number of sones, for example), which could result in the damaging effects.

## 7. Conclusions

There are clear benefits to assessing noise risk using binaural measurements and kurtosis-corrected SPL values. This approach can provide a more comprehensive assessment of the soundscape and the risks of excessive noise exposure and contribute to the prevention of asymmetrical hearing loss.

The results of this study can support initiatives to reduce occupational hearing loss in construction through a multifaceted research and outreach effort. This research has a potential to reach high-risk sectors, such as small employers, vulnerable workers, and residential and light commercial construction. The proposed findings of this research have several practical applications for both workers and their employers. First, the proposed technique can provide a much more reliable approach for measuring sound exposure, leading to a more efficient safety strategy in mitigating risk by reducing exposure time or using more-effective personal protective equipment. Second, using the proposed approach, a database of sound exposure for different construction activities can be developed to inform workplace scheduling and reassign at-risk workers. Finally, companies developing construction equipment and tools can benefit from the results of this study by obtaining a more accurate characterization of the sound generated by their products.

## Supplementary Material

Supplementary Material

## Figures and Tables

**Table 1.** Summary statistics.

		μ	σ	SE	95% CI for μ

Dosimeter	dBA	87.41	7.22	0.56	(86.308, 88.521)
Left Ear SPL	dBA	94.67	6.17	0.48	(93.721, 95.611)
Right Ear SPL	dBA	93.74	6.24	0.48	(92.786, 94.698)
Higher-SPL Ear	dBA	95.29	6.15	0.48	(94.348, 96.234)
Lower-SPL Ear	dBA	93.12	6.09	0.47	(92.183, 94.051)

Kurtosis, Left Ear	/	46.46	50.15	3.89	(38.78, 54.15)
Kurtosis, Right Ear	/	46.27	54.97	4.27	(37.85, 54.70)
Kurtosis Left Ear Correction	dB	6.70	2.35	0.18	(6.342, 7.061)
Kurtosis Right Ear Correction	dB	6.60	2.41	0.19	(6.230, 6.968)

Left Ear Kurtosis Corrected SPL	dBA	101.37	7.17	0.56	(100.268, 102.466)
Right Ear Kurtosis Corrected SPL	dBA	100.34	7.20	0.56	(99.237, 101.445)
Higher SPL Ear, After Kurtosis Correction	dBA	102.15	7.07	0.55	(101.065, 103.232)
Lower SPL Ear, After Kurtosis Correction	dBA	99.56	7.11	0.55	(98.471, 100.649)

**Table 2.** Hypothesis testing.

		μ_1_ – μ_2_	σ	SE	98% CI	*p*-Value
μ_1_: μ_2_:	Higher-SPL Ear Dosimeter	7.877	6.021	0.467	(6.779, 8.974)	<0.0001
μ_1_: μ_2_:	Left Ear Right Ear	0.924	2.495	0.194	(0.469, 1.378)	0.694
μ_1_: μ_2_:	Higher-SPL Ear Lower-SPL Ear	2.174	1.524	0.118	(1.897, 2.452)	<0.0001
Null hypothesis Alternative hypothesis		H_0_: μ_1_ – μ_2_ = 1 H_1_: μ_1_ – μ_2_ ≠ 1				

**Table 3.** Noise exposure in hours and the corresponding noise dose based on the noise SPL using NIOSH and OSHA calculation methods.

SPL (dBA)	NIOSH		OSHA	

	T (Hours)	Dose (%)	T (Hours)	Dose (%)

80.0	25.4	31	32.0	25
81.0	20.2	40	27.9	29
82.0	16.0	50	24.3	33
83.0	12.7	63	21.1	38
84.0	10.1	79	18.4	44
**85.0**	**8.0**	**100**	16.0	50
86.0	6.3	126	13.9	57
87.0	5.0	159	12.1	66
88.0	4.0	200	10.6	76
89.0	3.2	252	9.2	87
**90.0**	2.5	317	**8.0**	**100**
91.0	2.0	400	7.0	115
92.0	1.6	504	6.1	132

## Data Availability

Dataset is available on request from the authors.
