# Supplementary figures and images for: Construction Site Noise Exposure Assessment Using Binaural Measurements and Analysis
Source: Safety (Basel). Author manuscript; Available in PMC 2025 Feb 28. (PMC11870672; doi:10.3390/safety10040092)

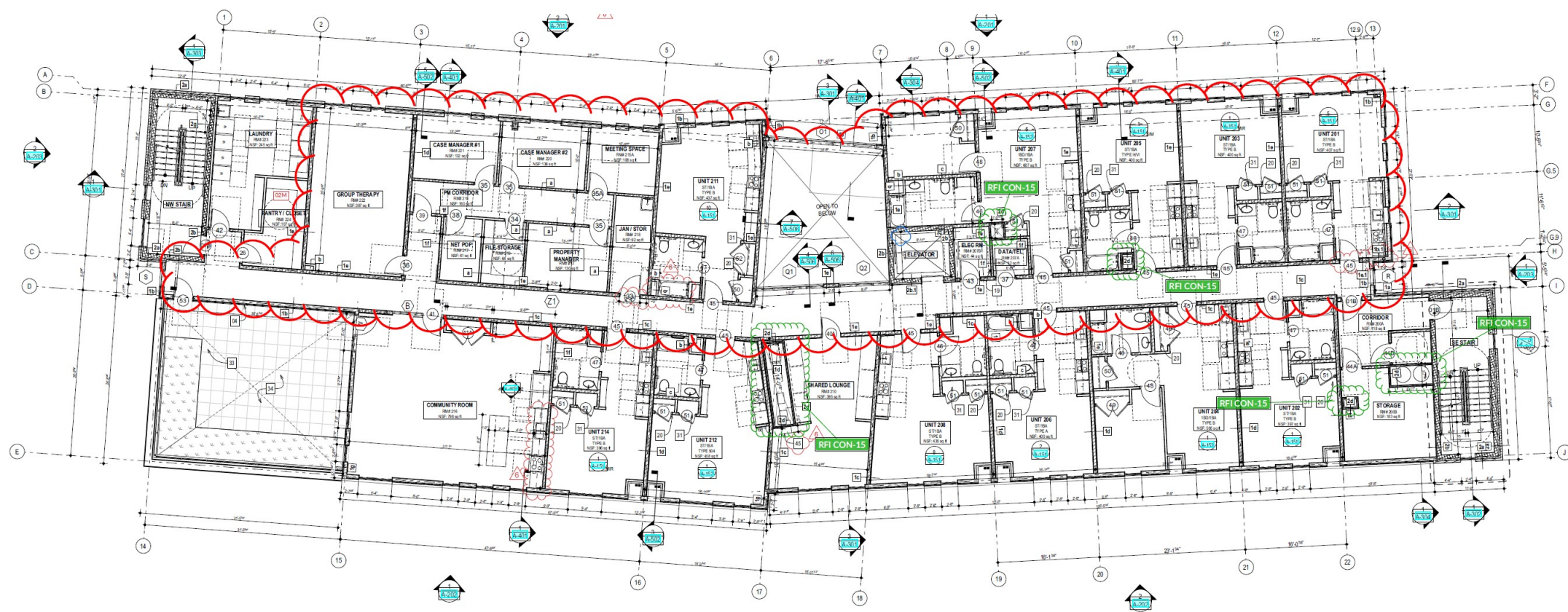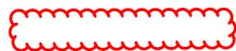

Work area of the construction workers

Supplement: Supplementary Material [file NIHMS2054969-supplement-Supplementary_Material.pdf]
